# Obesity in adolescents may be associated with limitations in daily activities and an increased level of anxiety in patients and their parents – preliminary results of a pilot study

**DOI:** 10.3389/fendo.2022.1007765

**Published:** 2022-10-07

**Authors:** Małgorzata Wójcik, Dawid Goncerz, Marta Piasny, Anna Surówka, Edyta Mazurek, Dorota Drożdż, Agnieszka Kozioł-Kozakowska, Jerzy B. Starzyk, Marta Makara-Studzińska

**Affiliations:** ^1^ Department of Pediatric and Adolescents Endocrinology, Chair of Pediatrics, Pediatric Institute, Jagiellonian University Medical College, Kraków, Poland; ^2^ Students’ Scientific Group, Department of Pediatric and Adolescents Endocrinology, Chair of Pediatrics, Pediatric Institute, Jagiellonian University Medical College, Kraków, Poland; ^3^ Department of Statistics, Faculty of Economics and Finance, Wroclaw University of Economics and Business, Wrocław, Poland; ^4^ Department of Pediatric Nephrology and Hypertension, Chair of Pediatrics, Pediatric Institute, Jagiellonian University Medical College, Kraków, Poland; ^5^ Department of Pediatrics, Gastroenterology and Nutrition, Jagiellonian University Medical College, Kraków, Poland; ^6^ Department of Health Psychology, Faculty of Health Science, Jagiellonian University, Medical College, Kraków, Poland

**Keywords:** obesity, depression, anxiety, adolescents, children, MAFLD

## Abstract

Obesity is a chronic disease, that in adolescents may lead to serious consequences affecting somatic and mental health. This study aimed to assess the prevalence of depressive symptoms and anxiety in adolescents with obesity and their parents. The relationships between depressive and anxiety symptoms and the somatic consequences of obesity were also analyzed.

**Material and Methods:**

19 patients with obesity (BMI Z-SCORE 2.1-5.5), at the age 16-17, and their parents answered validated questionnaires (Children’s Depression Inventory 2, The State-Trait Anxiety Inventory), and a survey assessing everyday functioning.

**Results:**

There were no significant differences in the occurrence of symptoms of depression in children and their parents: for the overall scale score of T-score (p=0.331), for the emotional problems (p=0.281) subscale, and the functional problems (p=0.147) subscale. The comparison of the results between boys and girls revealed no significant differences. A significantly higher level of anxiety was found in parents of children who gained weight in the year preceding the study (p = 0.046), and both in children and parents of children with metabolic-associated fatty liver disease – MAFLD (p=0.022 and p=0.007). According to adolescents, obesity affects the most leisure activities.

**Conclusion:**

Obesity, like any chronic disease, can have a significant impact on the emotional state of children and adolescents as well as the possibility of realizing interests and spending free time. Much more important than depressive disorders are anxiety disorders concerning both patients and their parents.

## Introduction

Obesity is a chronic disease that often occurs from early childhood, leading to the development of a cascade of other health problems (1). According to data published in 2017, the percentage of children with obesity increased eightfold between the years 1975 and 2016, and the total number in 2016 was 41 million (2). The negative impact of obesity on the somatic development of children and adolescents has been documented in many studies. It has been proven that even in the youngest age groups there is a risk of developing metabolic complications, such as arterial hypertension, liver steatosis, obstructive sleep apnea, vitamin D deficiency, and progressive disorders of glucose, lipid, and uric acid metabolism (3–5). Less is known about the association of obesity in the developmental age with mental health outcomes. The prevalence and future risk of anxiety, symptoms of depression, and everyday functioning in the family and school environment remain unclear (6).

The studies published so far and their meta-analyzes revealed that children and adolescents with obesity are at risk of the development of depressive symptoms or even overt depression, that may persist into adulthood (6, 7). That risk was higher for female patients (6, 8, 9). In some detailed studies, the authors also drew attention to disorders of the emotional state of adolescents with obesity other than depression, including anxiety. It has been shown that unrecognized and untreated emotional problems can lead to the development of mental health deterioration and eating disorders (10). To date, the relationship between the occurrence of metabolic complications of obesity and the occurrence of anxiety disorders, depressive symptoms, and everyday functioning in adolescents has not been analyzed. Some single studies have been conducted in adults, but the results are inconclusive (11, 12). Even less attention has been paid to the emotional problems of parents of obese children. Although the child’s disease, very often in the family context of obesity, can have a significant impact on parents’ emotional functioning (13). Parents of obese children seem to be at particular risk of developing anxiety and depression symptoms because, in addition to the treatment of a child with a chronic, difficult disease, they are often obese themselves.

This preliminary study aimed to assess the prevalence of depressive and anxiety symptoms in adolescents with obesity and their parents. The impact of medical factors, such as concomitant metabolic disorders, complications of obesity, and results of the body weight reduction on the symptoms of depression and anxiety, and selected aspects of everyday functioning were also analyzed.

## Material and methods

The preliminary study included 19 children (74% female), at the mean age of 16 (range 15-17 years) with obesity and their parents (14) The study used validated questionnaires (Children’s Depression Inventory 2, CDI2, and The State-Trait Anxiety Inventory, STAI, both in the Polish language version). These questionnaires were completed separately by the parent (n=19) and the patient. The method used to measure depression has a high-reliability coefficient. Cronbach’s α-coefficient measuring the reliability of the children’s and parents’ results for the tested sample and subscales ranged between 0.71 and 0.79, which proves that the scale is sufficiently reliable for the tested sample (15). The STAI questionnaire consists of two independent parts, each containing 20 statements. The part STAI X1 and STAI X2. X1 can evaluate anxiety considered as the current emotional state. This part of the questionnaire is a very sensitive tool. It enables one to trace the dynamics of anxiety even in short time intervals. X2, concerns anxiety understood as a personality trait (16). High levels of anxiety X1 and X2 defined has been defined for the value of the state scale: 8-10, and a high level of depressive symptoms was defined for the ten scale values above 64. Additionally, the participants of the study were asked about subjective feelings about the influence of the disease on key aspects of their lives: relationship with peers, school performance, way of spending free time, hobbies, and relations with siblings. The adolescents referred to each issue in the range from 1 to 5, where 1.2 meant that the disease did not affect the examined issues, and 4.5 - the disease’s impact on the examined factors. The middle answer was neutral and it was difficult to express the sentence. Such an answer was classified more as a positive answer, which in the case of this study means that the disease does not influence the examined issues. The study also analyzed the medical data regarding the occurrence of biochemical disorders typical of the metabolic syndrome (disorders of glucose metabolism, elevated triglycerides ≥ 1.7 mmol/L, and low HDL <1.0 mmol/L) (3), vitamin D deficiency, presence of biochemical and ultrasonographical presence of metabolic-associated fatty liver disease (MAFLD). The results of obesity treatment, defined as the change in body weight in the 12 months preceding the study, were also analyzed.

## Statistics

All statistical calculations were performed using the statistical package StatSoft Inc. STATISTICA (data analysis software system) version 13.3. The Shapiro-Wilk W test was used to check whether the quantitative variable was derived from a population with a normal distribution. The significance of differences between two groups in the model of unrelated variables was tested by Student’s t-test or Mann-Whitney U-test (when the assumptions of the parametric test were not met). The significance of differences between two groups in the model of related variables was tested by Student’s t-test or Wilcoxon’s test (when the assumptions of the parametric test were not met). Correlation analysis was used to determine the relationship, strength, and direction between ordinal variables by calculating the Spearman correlation coefficient. Fisher’s exact test was used to examine correlations for dichotomous characteristics, and Pearson’s chi-squared test for independence was used for categorical variables. The decision on the hypotheses to be verified was made by assuming a significance level of 0.05.

The odds ratio was used to assess the chance of an event occurring in one group to the chance of it occurring in another group.

## Results

The coefficient of age variation was 5.5% (less than 10%, not statistically significant). The mean BMI Z-SCORE was 3.5 (range 2.1-5.5) (14). In the study group, 74% of patients were already treated with the combined lifestyle intervention, including constant supervision by a dietician. Nevertheless, approximately 74% of participants in the year preceding the study increased their body weight. Odds ratio analysis showed, however, that dietician care increased the chance of losing weight almost six times compared to being left without such care. The most common concomitant problem was vitamin D deficiency (n=15; 79%). Impaired fasting glucose was diagnosed in one patient (5%), impaired glucose tolerance in 4 (21%), elevated triglycerides in 4 (21%), low HDL-cholesterol in 3 (16%), MAFLD features in 6 (32%) (see **Table 1**). The analysis of the responses showed that obesity has not been shown to affect relationships with peers. According to teenagers, obesity affects the most spending free time and pursuing a hobby. However, even for these variables, half of the respondents believe that the disease is not an obstacle in terms of leisure activities. Interestingly, almost 16% of respondents believe that obesity has a negative impact on school performance and the same number of young people believe that it limits the possibility of developing their interests (**Table 2**). There were no significant differences in the occurrence of symptoms of depression in children and their parents: for the overall scale score of T-score (p=0.331), for the emotional problems (p=0.281) subscale, and for the functional problems (p=0.147) subscale. The comparison of the results between boys and girls revealed no significant differences. There were also no significant differences in the level of depression (emotional problems, functional problems, and overall score CDI) between adolescents who increased their weight and those who lost weight (p = 0.103, p = 0.171; p = 0.085). The concomitant complications of obesity also were insignificant in terms of the occurrence of depression symptoms: impaired glucose tolerance (p=0.898, p=0.909; p=0.866); dyslipidemia (p=0.664, p=0.704; p=0.770), MAFLD (p=0.823, p=0.911; p=0.804), and vitamin D deficiency (p=0.921, p=0.203; p=0.471). The relationship between the results of the depression symptoms and BMI Z-SCORE, HOMA-IR, and vitamin D measured with the Spearman correlation was statically insignificant (p> 0.05). The results obtained from the STAI test allowed for the selection of participants with a high level of anxiety (a permanent trait - X2) and a high level of “transient” anxiety (X1). There were no significant differences in the distribution of sten X1 values between children and their parents (T=33.5; p=0.402). Significant differences were found in the distribution of the sten X2 values (T=17.0; p=0.008). There were no differences in anxiety (X1, X2 children, X2 parents) levels between boys and girls (p=0.546; p=0.738; p=0.212, respectively). However, a significantly higher level of X2 anxiety was found in parents of children who gained weight in the year preceding the study (p = 0.046). The chance of a high level of anxiety as a feature of X2 for parents of children who have increased their body weight was more than two times higher than for that who decreased weight. Statistically significant higher level of X1 and X2 parents was noticed in patients with MAFLD (p=0.022 and p=0.007). Additionally, the odds ratio analysis showed a more than ten times higher risk of a high level of anxiety (X2 children) in patients with MAFLD). The other concomitant complications of obesity were not significant for parameters of anxiety (X1, X2 children, X2 parents): impaired glucose tolerance (p=0.254, p=0.158; p=0.378); dyslipidemia (p=0.432, p=0.216; p=0.215), and vitamin D deficiency (p=0.803, p=0.794; p=0.582). The relationships between the anxiety levels X1, X2 children, X2 parents, BMI Z-SCORE, HOMA-IR, and vitamin D levels measured with the Spearman monotonic correlation coefficient were statically insignificant (p> 0.05).

**Table 1:** Characteristic of the study group.

	mean (range) [SD]	number (%) of te results out of range
Fasting glucose [mmol/L]	4.8 (3.6-5.8) [0.52]	1 (5%)
Glucose 120’ (oral glucose tolerance test) [mmol/L]	6.5 (4.8-9.4) [1.4]	4 (21%)
Triglicerydes [mmol/L]	1.27 (0.58-2.81) [0.57]	4 (21%)
HDL cholesterol [mmol/L]	1.2 (0.75-1.45) [0.22]	3 (16%)
25(OH)D [ng/mL]	19.95 (7.4-38) [9.7]	15 (79%)

**Table 2:** The influence of the disease on key aspects of adolescent’s lives.

variabile	Realtionship with peers		School performance		Way of spending free time		Hobby		Relations with siblings	
Answer (score 1-5)	*n_i_*	*ω_i_* [%]	*n_i_*	*ω_i_* [%]	*n_i_*	*ω_i_* [%]	*n_i_*	*ω_i_* [%]	*n_i_*	*ω_i_* [%]
1	7	3ω6.8	6	31.6	4	21.1	4	21.1	11	57.9
2	2	10.5	8	42.1	6	35.0	7	36.8	6	31.6
3	8	42.1	2	10.5	5	21.7	5	26.3	1	5.3
4	2	10.5	2	10.5	4	21.7	2	10.5	1	5.3
5			1	5.3			1	5.3		

## Discussion

Obesity itself can be a stressful state due to the high prevalence of weight stigma. This interaction between excessive body weight and emotional state may be bi-directional leading to a vicious cycle of stress and obesity (8, 17). In this study, we showed that according to adolescents with obesity, the disease most significantly limits the ability to pursue interests, hobbies, and leisure activities. Among obese adolescents diminished physical activity is a common problem, which itself leads to weight gain. It is worth noticing, that if patients with perceive obesity as a significant obstacle to physical activity, then putting pressure on them can cause frustration and even increase emotional problems. A detailed analysis of that problem was carried out by Jodkowska et al., obtaining similar results and concluding that in overcoming the barriers to physical activity in obese adolescents, one should aim to comprehensively reduce body weight and support health-oriented motivation (18). Contrary to most of the studies published so far, in this study we have not shown a significant predominance in the incidence of depressive symptoms in girls (6, 8, 9). Overall, in the present study, rates of depression in both adolescents and their parents were relatively low. Although it is commonly repeated, that obesity is related to depression, that thesis is not fully supported by research findings. Most of the studies relating depression to obesity in adolescents have generated inconsistent results. Among overweight children drawn from specialist clinics, approximately 24% are estimated to present symptoms of depression (19). However, it is worth noting that children and adolescents drawn from specialist clinics are not representative of children in the community and may overestimate the risk (20). In an excellent community-based study including over 6,000 participants, Wardle et al. showed that in adolescents, regardless of gender, socioeconomic status, or ethnicity, reports of depressive symptoms are not significantly higher in obese than normal-weight groups (21). In the population of adolescents with obesity, the problem of anxiety seems to be much more important. That issue should be analyzed in obese teenagers themselves and their parents. Although the results of the present study obtained in adolescents themselves did not show any significant correlation between treatment outcomes as measured by weight change and the occurrence of anxiety or depressive disorders, in the case of their parents, anxiety was more severe in those whose children did not lose weight. No similar, clear relationship was observed for depressive symptoms. However, the influence of a small number of participants on this result cannot be ruled out. It certainly requires further research. The problem of perceiving one’s own child’s obesity and the concerns about it has been analyzed by BeLue et al. Caregivers in this study consistently reported concern for their overweight children’s mental health and problem behavior (13). Obesity in adolescents may have consequences for caregivers and families, even if it is not a significant problem for the patients themselves. On this basis, one could speculate that reducing the child’s body weight, may be beneficial to reduce anxiety in parents. And as a further consequence, perhaps may improve the attitude towards treatment. In this context, the next observation from the present study concerning the role of a dietitian in the therapeutic process is of particular importance. Regular surveillance of the patient significantly increases the chance of losing weight. Similar results were obtained in the previous study (22). Therefore, it can be speculated that dietary habits may indirectly influence the psychological effects of treatment. This preliminary observation should become the subject of further research. Another important new observation in this study is the association of the severity of anxiety with the occurrence of organ complications of obesity, i.e. MAFLD. The risk of a high level of anxiety was 10 times higher in patients with MAFLD, than in those without that disorder. So far, the research has clearly distinguished between the somatic and psychological effects of obesity in adolescents. This analysis is the first attempt to combine both aspects. At present, we do not have sufficient evidence to show the direction of the relationship between a higher level of anxiety and the occurrence of MAFLD. Nevertheless, this observation is not entirely new. Some recent studies on the incidence of emotional, depressive, and anxiety disorders in adult patients with the liver disease found similar associations of incompletely explained origins (23). Some researchers have suggested the influence of chronic stress on the development of liver damage in people with obesity. According to this theory, as in type 2 diabetes and cardiovascular disease, stress involves both behavioral and biological responses, which activate the hypothalamic –pituitary – adrenal axis, resulting in elevated levels of cortisol and

pro-inflammatory biomarkers that could be involved in the development of MAFLD (24, 25). Also, pro-inflammatory pathways, immune dysregulation, and systemic or multi-organ inflammation are considered to mediate pathophysiological interaction between certain mental health disorders (25, 26). Finally, there are also established relationships between obesity and mental health that may be connected to changes in gut microbiota, resulting in such comorbidity. Recently, this mechanism of MAFLD development has also been suggested. According to this theory, perhaps both metabolic disorders and symptoms of anxiety and depression have a similar source in people with obesity (27). Undoubtedly further studies are needed to unravel the mechanism(s) of MAFLD and their psychological manifestations.

Although the results of the study turned out to be innovative and surprising, the work itself has significant limitations. The first one is the size of the group. As far as it is a pilot study with a small number of participants, unequal gender distribution, substantial variation in BMI Z-SCORE, and metabolic characteristics, the results need to be confirmed in a larger group of participants. Another is conducting a study in a clinic, on patients admitted to the hospital. A greater value of the results could be obtained by conducting the study in a larger community and comparing the results in people with obesity and normal body weight.

## Conclusions

Obesity, like any chronic disease, can have a significant impact on the emotional state of children and adolescents as well as the possibility of realizing interests and spending free time. Much more important than depressive disorders are anxiety disorders concerning both patients and their parents.

## Data availability statement

The raw data supporting the conclusions of this article will be made available by the authors, without undue reservation.

## Ethics statement

The study was approved by the local Jagiellonian University Ethics Committee (No 1072.6120.34.2021, date MAR-17-2021.). Written informed consent to participate in this study was provided by the participants’ legal guardian/next of kin.

## Author contributions

MW MM-S, DG, MP, AS, JS: conceived the project, DG, MP, AS, MW, DD, AK-K: collected data, DG, MW, MM-S, EM: wrote the original draft. DG, MW, MM-S, AK-K were responsible for conducting the systematic review. DG, EM performed the statistical analysis. JS: participated in revising the work for important intellectual content. All authors contributed to the article and approved the submitted version.

## Funding

This project is funded by POB qLIFE grant No. 1.012.996.2020

## Conflict of interest

The authors declare that the research was conducted in the absence of any commercial or financial relationships that could be construed as a potential conflict of interest.

## Publisher’s note

All claims expressed in this article are solely those of the authors and do not necessarily represent those of their affiliated organizations, or those of the publisher, the editors and the reviewers. Any product that may be evaluated in this article, or claim that may be made by its manufacturer, is not guaranteed or endorsed by the publisher.
